# First Principles Study of Bismuth Vacancy Formation in (111)-Strained BiFeO_3_

**DOI:** 10.3390/ma17225397

**Published:** 2024-11-05

**Authors:** Lu Xia, Thomas Tybell, Sverre M. Selbach

**Affiliations:** 1 Department of Material Science and Engineering, NTNU Norwegian University of Science and Technology, N-7491 Trondheim, Norway; 2 Department of Electronic Systems, NTNU Norwegian University of Science and Technology, N-7491 Trondheim, Norway

**Keywords:** bismuth ferrite, point defects, vacancies, epitaxial strain, multiferroic

## Abstract

Epitaxial strain is known to significantly influence the structural and functional properties of oxide thin films. However, its impact on point defect concentration has been less explored. Due to the challenges in experimentally measuring thin-film stoichiometry, computational studies become crucial. In this work, we use first-principles calculations based on density functional theory to investigate the formation and stability of Bi vacancies and Bi-O vacancy pairs in BiFeO_3_ (BFO) under (111) epitaxial strain. Our results demonstrate that compressive strain (−4%) decreases the formation enthalpy of Bi vacancies by 0.88 eV, whereas tensile strain (4%) increases it by 0.39 eV. Out-of-plane (OP) Bi-O vacancy pairs exhibit enhanced stability under both compressive and tensile strain, with formation enthalpy reductions of 1.49 eV and 1.05 eV, respectively. In contrast, in-plane (IP) vacancy pairs are stabilized under compressive strain but are insensitive to tensile strain. Finally, we discuss how these findings influence Bi stoichiometry during thin-film growth and the role of local strain fields in the formation of conducting domain walls.

## 1. Introduction

BiFeO_3_ (BFO) is a widely studied multiferroic material, exhibiting both ferroelectric and antiferromagnetic orders above room temperature (*T*_C_ = 830 °C, *T*_N_ = 370 °C), along with magnetoelectric coupling. Its large ferroelectric polarization (~90 µC/cm^2^), primarily driven by the displacement of Bi^3+^ along the [111] pseudocubic axis [1], makes BFO a candidate for device applications such as sensors, actuators, and memory devices [2,3]. The majority of technological applications of BFO rely on thin films [4,5]. Perovskite structures with rhombohedral space group *R*3*c* are readily grown as high-quality epitaxial thin films on a variety of different substrates. The resulting epitaxial strain leads to improved ferroelectric, piezoelectric and magnetic properties [6,7,8,9,10]. Although the structural effects of epitaxial strain are well understood [11,12], the influence of strain on compositional degrees of freedom, such as cation and anion vacancies, has received comparatively less attention [13,14]. This oversight is partly due to the difficulty of measuring subtle stoichiometric variations at the nanoscale [15,16].

Given that bismuth (Bi) is a volatile element and plays a critical role in determining the physical properties of BiFeO_3_, understanding the effects of epitaxial strain on Bi vacancies is crucial. Recent research has shown that Bi volatility during film growth can lead to non-stoichiometry, influencing both ferroelectric and magnetic behaviors [17,18]. Specifically, local Bi deficiency has been linked to the formation of conductive domain walls, which have *p*-type conductivity and are significantly influenced by the epitaxial strain present during film growth [19,20]. Motivated by the observed dependence of vacancy formation on strain in other oxide systems [21,22], we investigated the formation of Bi and Bi-O vacancy pairs in (111)-strained BFO using DFT calculations. This orientation is of particular interest for device applications due to the out-of-plane alignment of ferroelectric polarization, providing a direct link between polarization and strain-driven defect formation. However, growing (111)-oriented thin perovskite films is considerably more challenging [23] than growing films in the more traditional (001)-orientation, calling for a better understanding of how strain in the (111)-plane affects vacancy formation and local stoichiometry.

## 2. Computational Methods

**Figure 1:**
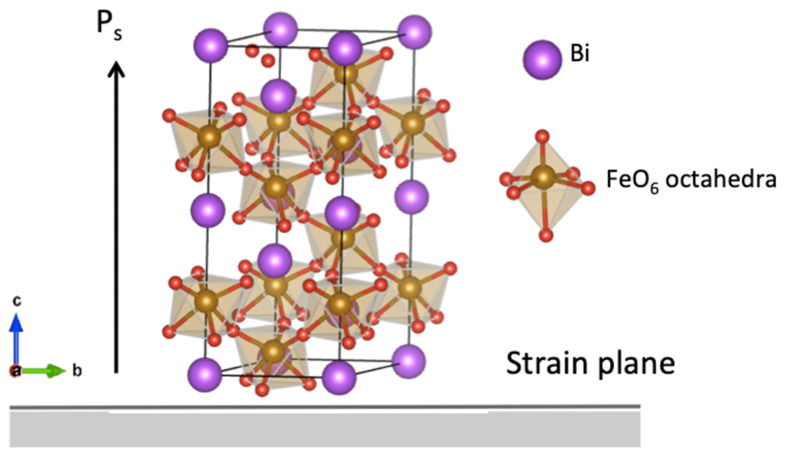
The unit cell of BiFeO_3_ in a hexagonal setting with spontaneous polarization P_s_ is in the [001] (c-axis) direction, which is parallel to the [111] direction in the pseudo-cubic unit cell.

Density Functional Theory (DFT) calculations were performed with the projector-augmented wave (PAW) method [24] as implemented in the Vienna Ab initio Simulation Package (VASP 6.x) [25,26] We employed the GGA+U approximation [27,28] with the PBEsol functional [29], using Bi_d, Fe_pv and standard O PBE PAW potentials, and set the plane wave cutoff energy at 550 eV. Collinear G-type antiferromagnetism was imposed on the Fe sublattice, with a Hubbard U of 4 eV applied to the Fe 3*d* states [11]. Defect calculations were performed on a 120-atom supercell (2 × 2 × 1 of the 30-atom trigonal unit cell) with a gamma-centered 2 × 2 × 2 k-point mesh for reciprocal space integration. The spontaneous polarization is out-of-plane along the [111] pseudocubic direction, as illustrated in Figure 1.

**Figure 2:**
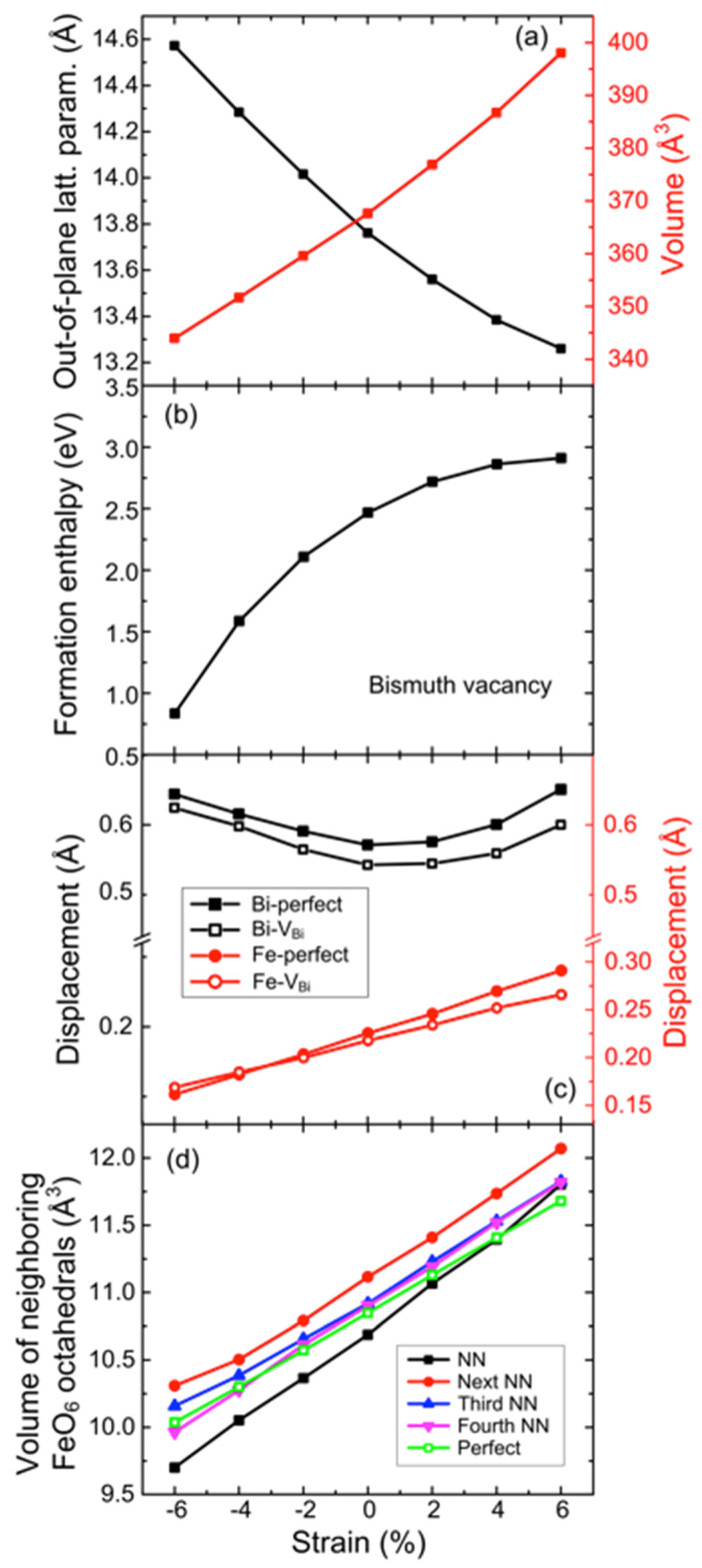
(**a**) Out-of-plane lattice parameter and primitive unit cell volume of BiFeO_3_ under (111) epitaxial strain. (**b**) Formation enthalpy of a Bi vacancy (V_Bi_), (**c**) polar out-of-plane displacements of Bi and Fe from their centrosymmetric reference positions for stoichiometric and Bi deficient cells, and (**d**) polyhedral volumes for the FeO_6_ octahedra surrounding the V_Bi_ as a function of (111) epitaxial strain.

Epitaxial strain in the (111) plane was simulated by fixing the in-plane lattice parameters to strain values defined as (a_h_ − a_h,0_)/a_h,0_ * 100%, where a_h,0_ is the unstrained equilibrium in-plane lattice parameter. The out-of-plane lattice parameter c and atomic positions were relaxed until the forces on the ions were less than 0.01 eV/Å, and the resulting out-of-plane lattice parameter and unit cell volume for stoichiometric material is presented in Figure 2a. This setup allows us to accurately simulate the effects of strain on vacancy formation while avoiding complications from structural phase transitions.

## 3. Results and Discussion

In this section, we present and discuss the results of our first-principles calculation for BiFeO_3_ thin films under (111) epitaxial strain. Our investigation focuses on the formation of Bi vacancies (V_Bi_) and Bi-O vacancy pairs (V_Bi_-V_O_) and how strain affects their stability, electronic structure and overall impact on the material’s ferroelectric and magnetic properties. We structure this discussion sequentially, beginning with vacancy formation and strain effects, followed by an examination of the electronic structure and, finally, implications for real-world applications. 

### 3.1. Bismuth Vacancies with Electronic Charge Compensation

The formation of bismuth vacancies was investigated by removing a single Bi atom from a 120-atom supercell, corresponding to a vacancy concentration of 4.2% (Bi_0.958_FeO_3_). Although this defect concentration is relatively high, it is within the range of experimental conditions [30]. Our analysis here focuses on electronic charge compensation, which occurs under oxygen-rich conditions. In this scenario, the charge from a single V_Bi_ is compensated by the oxidation of three Fe^3+^ ions to Fe^4+^, generating three holes. The formation energy of a $V_{\mathrm{Bi}}$, denoted as $E_{V_{\mathrm{Bi}}}$, for a neutral cell is calculated using the following equation:

(1) $$E_{V_{\mathrm{Bi}}}=E_{tot,V_{\mathrm{Bi}}}-E_{tot,stoich}+\mu_{\mathrm{Bi}}$$

where $E_{tot,V_{\mathrm{Bi}}}$ and $E_{tot,stoich}$ are the total energies of the defect-containing cell and the stoichiometric (perfect) cell, respectively. The chemical potential of Bi, $\mu_{\mathrm{Bi}}$, is introduced to maintain mass balance and is explained in detail in the Supplementary Materials. The formation energy of V_Bi_ under oxygen-rich conditions in unstrained bulk BFO was calculated to be 2.46 eV, increasing to 5.54 eV under oxygen-poor conditions, in agreement with previously theoretical studies [31]. Strain has a significant impact on Bi vacancy stability. Compressive strain strongly stabilizes Bi vacnacies, with formation energy decreasing by 0.88 eV as strain increases from 0 to 4%, exhibiting monotonic, non-linear scaling (Figure 2b). Conversely, tensile strain destabilizes V_Bi_, increasing the formation energy by 0.39 eV for a strain of +4%.

The strain sensitivity of Bi vacancy formation can be understood as the inverse of the lattice contraction that occurs upon vacancy formation in bulk material. The full geometry optimization of an unstrained Bi_23_Fe_24_O_72_ supercell reveals a volume contraction of 0.56%, which can be considered negative chemical expansion [32]. The charge-compensating oxidation of Fe^3+^ (0.645 Å, C.N. = 6) to Fe^4+^ (0.585 Å, C.N. = 6) further contributes to lattice contraction. Although the removal of a Bi^3+^ cation reduces the local shielding of repulsive forces between O^2−^ anions, the overall effect of V_Bi_ formation is lattice contraction. As a result, bismuth vacancy formation alleviates compressive strain, while tensile strain suppresses Bi vacancy formation.

### 3.2. Bismuth Vacancies with Ionic Charge Compensation

Under oxygen-poor conditions, Bi vacancy is compensated not by electronic holes but by the formation of oxygen vacancies. In this case, we consider an intermediate scenario where one Bi vacancy is charge-compensated by one hole and one oxygen vacancy, leading to mild hole doping. The resulting supercell stoichiometry becomes Bi_0.958_FeO_2.958_. The mechanism can be described by the following reaction:

(2) $${\mathrm{BiFeO}}_{3}\;\to \;{\mathrm{Bi}}_{1-x}{\mathrm{Fe}}_{1-x}^{3+}{\mathrm{Fe}}_{x}^{4+}O_{3-\delta }+\mathrm{xBi}\left(g\right)+x/2\;O_{2}\left(g\right)\;$$

In Kröger–Vink notation, this reaction is equivalent to

(3) $$1/2\;{\mathrm{Fe}}_{2}O_{3}+1/4\;O_{2}\left(g\right)\overset{{\mathrm{BiFeO}}_{3}}{\to }V_{\mathrm{Bi}}^{\prime \prime \prime }+{\mathrm{Fe}}_{\mathrm{Fe}}^{\cdot }+2O_{O}^{x}+V_{O}^{\cdot \cdot }$$

**Figure 3:**
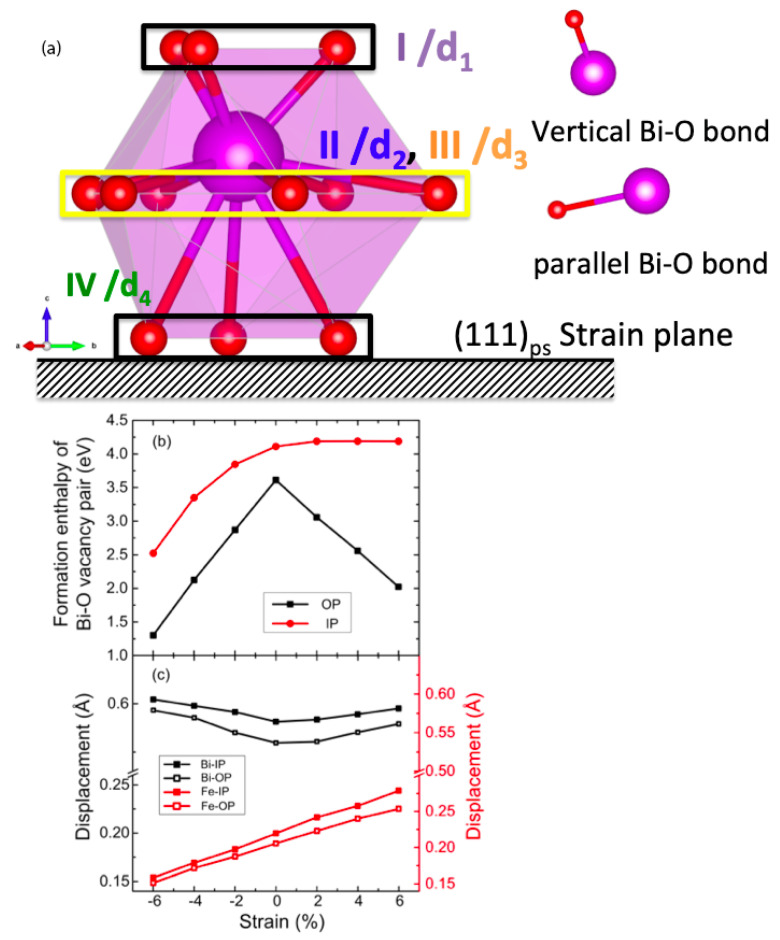
(**a**) Schematic illustration of two different types of Bi-O vacancy pairs relative to the epitaxial strain plane; (**b**) the formation energy of Bi-O vacancy pairs (Type I and Type III) as a function of strain; (**c**) polar out-of-plane displacements of Bi (black) and Fe (red) from their centrosymmetric reference positions for cells, including in-plane and out-of-plane Bi-O vacancy pairs.

Here, we investigated four types of Bi-O vacancy pairs, depending on their relative orientations to the strain plane as well as the distance between the two missing atoms. The different vacancy pairs are illustrated in Figure 3a. The formation enthalpies calculated for four types of Bi-O vacancy pairs in unstrained bulk BFO are detailed in Table 1.

V_Bi_-V_O_ pairs that are orientated relatively parallel or perpendicular to the (111) strain plane are labeled as ‘in-plane’ (IP) and out-of-plane (OP), respectively. We found that both the orientation and the distance between missing atoms in the perfect structure play a critical role in determining the stability of Bi-O vacancy pairs. For shorter vacancy pairs, the OP orientation is favored over the IP configuration, similar to the preferred alignment of V_Pb_-V_O_ vacancy pairs in tetragonal PbTiO_3_, which form so-called symmetry-conforming defects [33]. In contrast, no such orientation preference is observed for longer vacancy pairs. In addition, IP vacancy pairs are generally less stable than OP pairs but are more sensitive to the distance between the missing atoms. Shorter IP vacancy pairs are favored over longer ones by approximately 0.2 eV.

Furthermore, IP and OP vacancy pairs respond differently to strain (Figure 3b); OP pairs remain the most stable across all strain values, with their formation energy significantly reduced by both compressive and tensile strain: 1.49 eV under 4% compressive strain and 1.05 eV under 4% tensile strain. In contrast, IP pairs are primarily stabilized by compressive strain, with a reduction in formation energy of 0.76 eV from 0% to 4%. Tensile strain, however, weakly destabilizes IP pairs, with a small increase in formation energy of 0.08 eV.

As discussed previously, the stabilization of V_Bi_ under compressive strain can be attributed to the mitigation of lattice contraction and the removal of large Bi^3+^ cations. However, the stabilization of V_Bi_-V_O_ pairs under both compressive and tensile strain is less straightforward, due to the competing effects at play; both the removal of Bi^3+^ and the oxidation of Fe^3+^ to Fe^4+^ promote lattice contraction, while the formation of oxygen vacancies tends to favor lattice expansion.

### 3.3. Structural Changes and Polarization

The out-of-plane axis expands and contracts under compressive and tensile strain, respectively, with a net volume increase under tensile strain, as shown in Figure 2a. In addition, the introduction of V_Bi_ and V_Bi_-V_O_ pairs leads to significant structural changes in the surrounding lattice. In unstrained stoichiometric BFO, Fe-O bonds have two distinct lengths, 1.96 Å and 2.09 Å, resulting in an octahedral volume of 10.84 Å^3^. Upon Bi vacancy formation, the nearest-neighbor FeO_6_ octahedra contract to 10.67 Å^3^ due to both the removal of Bi^3+^ and the partial oxidation of Fe^3+^ to Fe^4+^. Meanwhile, second-neighbor octahedra expand to 11.12 Å^3^ to accommodate the local strain caused by the contraction around Fe^4+^ (Figure 2d). This localized distortion indicates a relatively short structural screening length of approximately 8 Å, consistent with previous observations of strain-induced lattice distortion [32]. 

Epitaxial strain further enhances the polar displacements of Bi and Fe from their centrosymmetric positions. Compressive strain increases Bi displacements, while Fe displacements increase monotonically from compressive to tensile strain. Interestingly, the presence of V_Bi_ reduces Bi displacement, particularly under tensile strain, but leaves Fe displacements largely unaffected (Figure 2c and Figure 3c). Despite these local distortions, the overall ferroelectric polarization remains largely unchanged, indicating that BFO can maintain its ferroelectric properties even with significant Bi vacancy concentration. 

### 3.4. Electronic Structure

**Figure 4:**
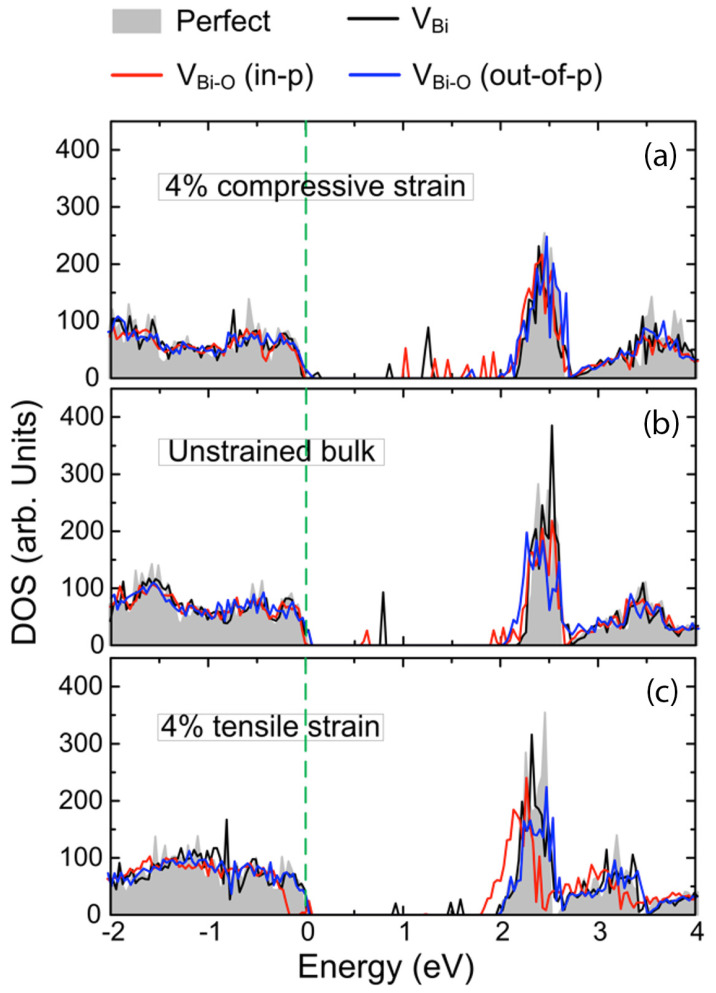
Total DOS of stoichiometric BFO, BFO with one Bi vacancy, and with in-plane and out-of-plane Bi-O vacancy pairs under (**a**) 4% compressive strain, (**b**) zero strain and (**c**) 4% tensile strain, respectively.

**Figure 5:**
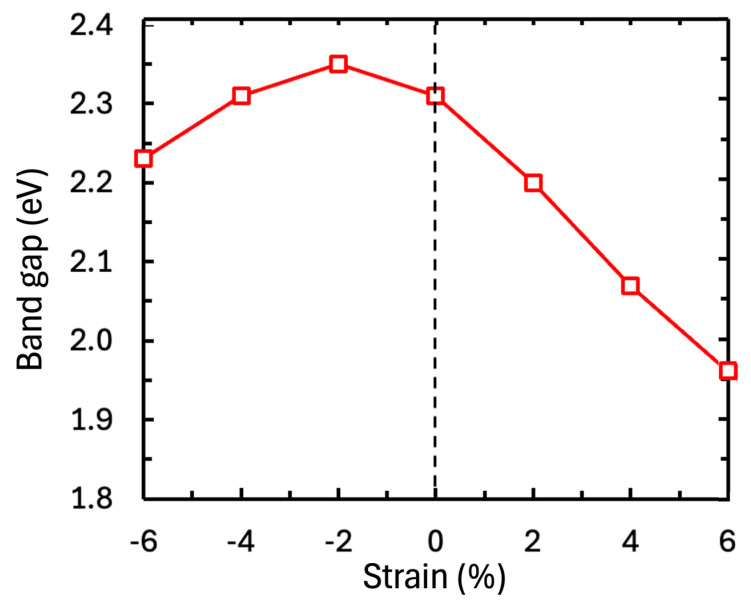
Electronic band gap of stoichiometric (111)-oriented BiFeO_3_ as a function of biaxial strain.

The electronic density of states (DOS) for stoichiometric and vacancy-containing BFO thin films presented in Figure 4 provides further insights into how strain influences the material’s electronic properties. For stoichiometric BFO, the band gap is calculated to be 2.3 eV, with the top of the valence band dominated by O 2p states and the bottom of the conduction band by Fe 3d states [34]. Under tensile strain, the band gap narrows to 2 eV at 4% strain, while compressive strain causes only minor changes. In cells containing Bi vacancies, the charge-compensating oxidation of Fe causes a portion of the Fe 3d states to shift into the bad gap, creating gap states with Fe 3d-O 2d character. These defect states are located just below the Fermi Energy, suggesting that Bi vacancies induce *p*-type conductivity, consistent with experimental observations of bulk BFO [35] and conducting domain walls [19]. The gap states induced by Bi vacancies and Bi-O vacancy pairs dominate the electronic structure and band gap changes compared to the isolated effect of strain on the stoichiometric cells, as illustrated in Figure 5. The electronic band gap is relatively insensitive to compressive strain, while in contrast, tensile strain significantly reduces the band gap from 2.31 eV to 2.20, 2.07 and 1.96 eV at 2, 4 and 6% strain, respectively. The shift in absolute values of the valence band maximum as a function of strain is illustrated in Supplementary Figure S1.

### 3.5. Implications for Thin Film Synthesis and Domain Walls

Our results suggest that BiFeO_3_ thin films will become increasingly susceptible to Bi deficiency as the strain magnitude increases, except under tensile strain in oxygen-rich conditions, where V_Bi_ formation is somewhat suppressed. Therefore, the likelihood of Bi loss during thin-film growth and processing increases with higher strain magnitudes, particularly under inert or reducing conditions. In addition to the more readily observable changes in crystal symmetry by diffraction methods, cation vacancy formation should also be anticipated in epitaxial films subjected to greater epitaxial strain [36].

These findings may also help explain reports of Bi-deficient ferroelectric domain walls in BFO [19]. Domain walls inherently induce local strain fields due to the discontinuities in polarization direction. Our results on coherently strained BFO strongly suggest that bismuth vacancies are also more likely to form in the strained local structure in the vicinity of domain walls. Furthermore, strain lowering of V_Bi_ formation energy also implies that domain walls can act as sinks for diffusion Bi vacancies during high-temperature annealing below the Curie temperature (*T*_C_), in agreement with the experimental findings of Rojac et al. [19].

## 4. Conclusions

In this study, we used DFT calculations to investigate the stability of Bi vacancies and Bi-O vacancy pairs as a function of epitaxial strain in (111)-oriented BiFeO_3_ (BFO) thin films. Our results show that Bi vacancies in charge-neutral cells are more energetically favorable under compressive strain than tensile strain when charge compensation occurs through the oxidation of Fe (no oxygen vacancy). Bi-O vacancy pairs aligned out-of-plane with respect to the (111) strain plane exhibit enhanced stability under both compressive and tensile strain, whereas in-plane Bi-O vacancy pairs are stabilized by compressive strain but remain relatively insensitive to tensile strain. Out-of-plane vacancy pairs, which are more aligned with the polarization direction, are favored over in-plane pairs for all strain values investigated. The promotion of Bi vacancies, with or without accompanying oxygen vacancies, is particularly relevant for thin film growth, as strain tends to enhance Bi deficiency. Finally, we expect the local chemical composition around ferroelectric domain walls to be Bi-deficient due to the localized strain fields surrounding these regions.

## Figures and Tables

**Table 1:** The formation enthalpies of different Bi-O vacancy pairs in unstrained bulk BiFeO_3_.

Type	Orientation of V_Bi_-V_O_ Pair	V_Bi_-V_O_ Distance (Å)	Formation Enthalpy of Vacancy Pair (eV)
I	OP	2.32	3.60
II	OP	3.35	3.68
III	IP	2.45	4.19
IV	IP	3.23	3.97

## Data Availability

The original contributions presented in the study are included in the article/Supplementary Material, further inquiries can be directed to the corresponding author.

## Supplementary Materials

The following supporting information can be downloaded at: https://www.mdpi.com/article/10.3390/ma17225397/s1, Table S1: Results from strained stoichiometric cells. Table S2: The chemical potentials of Bi, Fe and O. Figure S1: Electronic band edges as a function of strain. Figure S2: Formation enthalpy of bismuth vacancy.

## References

[1] Catalan G., Scott J.F. (2009). Physics and Applications of Bismuth Ferrite. Adv. Mater..

[2] Hemme P., Philippe J.-C., Medeiros A., Alekhin A., Houver S., Gallais Y., Sacuto A., Forget A., Colson D., Mantri S. (2023). Tuning the multiferroic properties of BiFeO_3_ under uniaxial strain. Phys. Rev. Lett..

[3] Prakash C., Yadav A.K., Dixit A. (2023). Low power highly flexible BiFeO_3_-based resistive random access memory (RRAM) with the coexistence of negative differential resistance (NDR). Phys. Chem. Chem. Phys..

[4] Ramesh R., Spaldin N.A. (2007). Multiferroics: Progress and prospects in thin films. Nat. Mater..

[5] Martin L.W., Rappe A.M. (2016). Thin-film ferroelectric materials and their applications. Nat. Rev. Mater..

[6] Wang J., Neaton J.B., Zheng H., Nagarajan V., Ogale S.B., Liu B., Viehland D., Vaithyanathan V., Schlom D.G., Waghmare U.V. (2003). Epitaxial BiFeO_3_ Multiferroic Thin Film Heterostructures. Science.

[7] Li J., Wang J., Wuttig M., Ramesh R., Wang N., Ruette B., Pyatakov A.P., Zvezdin A.K., Viehland D. (2004). Dramatically enhanced polarization in (001), (101), and (111) BiFeO_3_ thin films due to epitiaxial-induced transitions. Appl. Phys. Lett..

[8] Jang H.W., Ortiz D., Baek S., Folkman C.M., Das R.R., Shafer P., Chen Y., Nelson C.T., Pan X., Ramesh R. (2009). Domain Engineering for Enhanced Ferroelectric Properties of Epitaxial (001) BiFeO Thin Films. Adv. Mater..

[9] Infante I.C., Lisenkov S., Dupe B., Bibes M., Fusil S., Jacquet E., Geneste G., Petit S., Courtial A., Juraszek J. (2010). Bridging multiferroic phase transitions by epitaxial strain in BiFeO_3_. Phys. Rev. Lett..

[10] Zeches R.J., Rossell M.D., Zhang J.X., Hatt A.J., He Q., Yang C.H., Kumar A., Wang C.H., Melville A., Adamo C. (2009). A strain-driven morphotropic phase boundary in BiFeO_3_. Science.

[11] Hatt A.J., Spaldin N.A. (2010). Ederer, Strain-induced isosymmetric phase ransition in BiFeO_3_. Phys. Rev. B.

[12] Béa H., Dupé B., Fusil S., Mattana R., Jacquet E., Warot-Fonrose B., Wilhelm F., Rogalev A., Petit S., Cros V. (2009). Evidence for Room-Temperature Multiferroicity in a Compound with a Giant Axial Ratio. Phys. Rev. Lett..

[13] Dedon L.R., Saremi S., Chen Z.H., Damodaran A.R., Apgar B.A., Gao R., Martin L.W. (2016). Nonstoichiometry, structure, and properties of BiFeO_3_ films. Chem. Mater..

[14] Lahmar A., Zhao K., Habouti S., Dietze M., Solterbeck C.-H., Es-Souni M. (2011). Off-stoichiometry effects on BiFeO_3_ thin films. Solid State Ionics.

[15] Kim Y.M., Morozovska A., Eliseev E., Oxley M.P., Mishra R., Selbach S.M., Grande T., Pantelides S.T., Kalinin S.V., Borisevich A.Y. (2014). Direct observation of ferroelectric field effect and vacancy-controlled screening at the BiFeO_3_/LaxSr1−xMnO_3_ interface. Nat. Mater..

[16] Kalinin S.V., Spaldin N.A. (2013). Functional Ion Defects in Transition Metal Oxides. Science.

[17] Yuan H., Pal S., Forrester C., He Q., Briscoe J. (2024). Understanding the impact of Bi stoichiometry towards optimized BiFeO_3_ photocathodes: Structure, mor-phology, defects and ferroelectricity. J. Mater. Chem. A Mater..

[18] Prasad N.P., Rohnke M., Verheijen M.A., Sturm J.M., Hofmann J.P., Hensen E.J.M., Bieberle-Hutter A. (2023). Role of excess Bi on the properties and per-formance of BiFeO_3_ thin-film photocathodes. ACS Appl. Energy. Mater..

[19] Rojac T., Bencan A., Drazic G., Sakamoto N., Ursic H., Jancar B., Tavcar G., Makarovic M., Walker J., Malic B. (2016). Domain-wall conduction in ferroelectric BiFeO_3_ controlled by accumulation of charged defects. Nat. Mater..

[20] Seidel J., Martin L.W., He Q., Zhan Q., Chu Y.H., Rother A., Hawkridge M.E., Maksymovych P., Yu P., Gajek M. (2009). Conduction at domain walls in oxide multiferroics. Nat. Mater..

[21] Aschauer U., Pfenninger R., Selbach S.M., Grande T., Spaldin N.A. (2013). Strain-controlled oxygen vacancy formation and ordering in CaMnO_3_. Phys. Rev. B.

[22] Aschauer U., Vonrüti N., Spaldin N.A. (2015). Effect of epitaxial strain on cation and anion vacancy formation in MnO. Phys. Rev. B.

[23] Hallsteinsen I., Boschker J.E., Nord M., Lee S., Rzchowski M., Vullum P.E., Grepstad J.K., Holmestad R., Eom C.B., Tybell T. (2013). Surface stability of epitaxial La0.7Sr0.3MnO3 thin films on (111)-oriented SrTiO_3_. J. Appl. Phys..

[24] Blöchl P.E. (1994). Projector augmented-wave method. Phys. Rev. B.

[25] Kresse G., Joubert D. (1999). From ultrasoft pseudopotentials to the projector augmented-wave method. Phys. Rev. B.

[26] Kresse G., Furthmuller J. (1996). Efficient iterative schemes for ab initio total-energy calculations using a plane-wave basis set. Phys. Rev. B.

[27] Anisimov V.I., Zaanen J., Andersen O.K. (1991). Band theory and Mott insulators: Hubbard U instead of Stoner I. Phys. Rev. B.

[28] Dudarev S.L., Botton G.A., Savrasov S.Y., Humphreys C.J., Sutton A.P. (1998). Electron-energy-loss spectra and the structural stability of nickel oxide: An LSDA+U study. Phys. Rev. B.

[29] Perdew J.P., Ruzsinszky A., Csonka G.I., Vydrov O.A., Scuseria G.E., Constantin L.A., Zhou X., Burke K. (2008). Restoring the Density-Gradient Expansion for Exchange in Solids and Surfaces. Phys. Rev. Lett..

[30] Chen J.Y., Wang Y., Wang H., Zhang S.M., Deng Y. (2016). Bi deficiency-tuned functionality in multiferroic Bi_1_-δFe_0.95_Mn_0.05_O_3_ films. Sci. Rep..

[31] Zhang Z., Wu P., Chen L., Wang J. (2010). Density functional theory plus U study of vacancy formations in bismuth ferrite. Appl. Phys. Lett..

[32] Ren X. (2004). Large electric-field-induced strain in ferroelectric crystals by point-defect-mediated reversible domain switching. Nat. Mater..

[33] Eichel R.A., Erhart P., Traskelin P., Albe K., Kungl H., Hoffmann M.J. (2008). Defect-dipole formation in copper-doped PbTiO3 ferroelectrics. Phys. Rev. Lett..

[34] Paudel T.R., Jaswal S.S., Tsymbal E.Y. (2012). Intrinsic defects in multiferroic BiFeO_3_ and their effect on magnetism. Phys. Rev. B.

[35] Wefring E.T., Einarsrud M.-A. (2015). Grande, Electrical conductivity and thermopower of (1-x)BiFeO_3_–xBi0.5K0.5TiO_3_ (x=0.1, 0.2) ceramics near the ferroelec-tric to paraelectric phase transition. Phys. Chem. Chem. Phys..

[36] Xia L., Tybell T., Selbach S.M. (2019). Bi vacancy formation in BiFeO_3_ epitaxial thin films under compressive (001)-strain from first principles. J. Mater. Chem. C.

