# Supplementary material for: First Principles Study of Bismuth Vacancy Formation in (111)-Strained BiFeO3
Source: Materials (Basel). 2024 Nov 5;17(22):5397. doi: 10.3390/ma17225397 (PMC11595943; doi:10.3390/ma17225397)
Supplement: Supplementary file 1 [file materials-17-05397-s001.zip › materials-3244268-supplementary.pdf]

Supplementary Information for:

Bismuth vacancy formation in (111)-strained $BiFeO_3$

Lu Xia[1], Thomas Tybell[2], Sverre M. Selbach[1,a)]

[1]Department of Material Science and Engineering, NTNU Norwegian University of Science and Technology, N-7491 Trondheim, Norway
[2]Department of Electronic Systems, NTNU Norwegian University of Science and Technology, N-7491 Trondheim, Norway
[a)]Corresponding author (e-mail): selbach@ntnu.no

Results from stoichiometric cells

The lattice parameters of relaxed stoichiometric cells and polarizations calculated by Berry phase method are given in Table S1. Our simulations show that polarization is insensitive to strain.

TABLE S1. Strain ε, in-plane lattice parameter a, relaxed out-of-plane lattice parameter c, volume V of the hexagonal unit cell (containing six formula units), spontaneous polarization P_s calculated by Berry-phase method, the polarization P_s* calculated by point charge model, the ratio of P_s* and P_s.

ε (%)	a (Å)	c (Å)	V (Å)	P_s (μC/cm^2)
-6	5.221	14.572	343.969	93.9
-4	5.332	14.284	351.680	92.7
-2	5.443	14.016	359.587	92.5
0	5.554	13.764	367.622	92.6
2	5.665	13.560	376.889	93.0
4	5.776	13.385	386.740	94.4
6	5.887	13.260	398.024	97.4

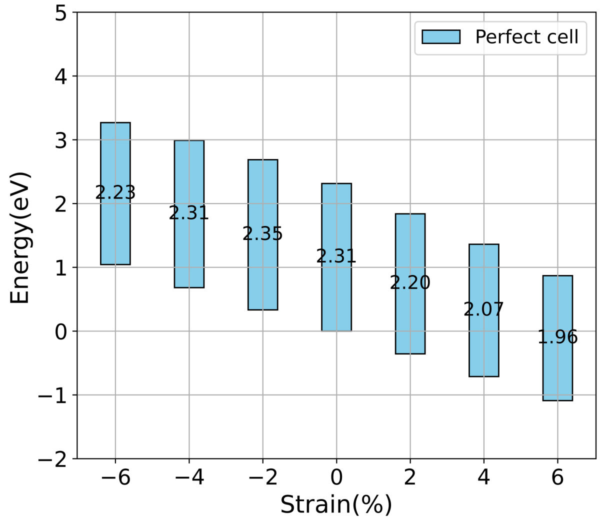

Figure S1. Electronic band edges and band gaps of stoichiometric $BiFeO_3$ as a function of (111)-strain. The valence band maximum is set to 0 for 0% strain as a reference value.

Chemical potentials:

Determination of μ values needs to establish different chemical reactions of the constitute oxides of BFO. In order to do that, we firstly performed calculations for many reference materials of their total energies (chemical potentials), i.e., Bi (rhombohedral, R-3m, space group 166), Fe (cubic, Im-3m, space group 229), Bi_2O_3 (monoclinic, P21/c, space group 14), FeO (cubic, Fm-3m, space group 225), Fe_3O_4 (cubic, Fd-3m, space group 227), Fe_2O_3 (rhombohedral, R-3c, space group 167) and O_2 molecular. For this letter, the oxygen rich condition is defined using equilibrium of $O_2/Fe_2O_3/BiFeO_3$, while the oxygen poor condition is defined by equilibrium of $Bi/Bi_2O_3/BiFeO_3$. The total energies of the reference materials were obtained after relaxation with the same computational method for consistency and the chemical potentials of Bi, Fe and O used in this letter are listed in Table S2. The Bi vacancy formation energy as a function of oxygen chemical potential is illustrated in Fig. S1.

TABLE S2. The chemical potentials of Bi, Fe and O

Chemical condition	Chemical potential (eV)		
	μ_{Bi}	μ_{Fe}	μ_O
Oxygen-rich<br>$\mu_{Bi} = (2\mu_{BiFeO_3} - \mu_{Fe_2O_3} - 3\mu_O)/2$<br>$\mu_{Fe} = (\mu_{Fe_2O_3} - 3\mu_O)/2$<br>$\mu_O = \mu_O$	-7.54	-10.5	-5.41
Oxygen-poor<br>$\mu_{Bi} = \mu_{Bi}$<br>$\mu_{Fe} = [\mu_{BiFeO_3} - \mu_{Bi_2O_3} + \mu_{Bi}]$<br>$\mu_O = [\mu_{Bi_2O_3} - 2\mu_{Bi}]/3$	-4.31	-7.27	-7.29

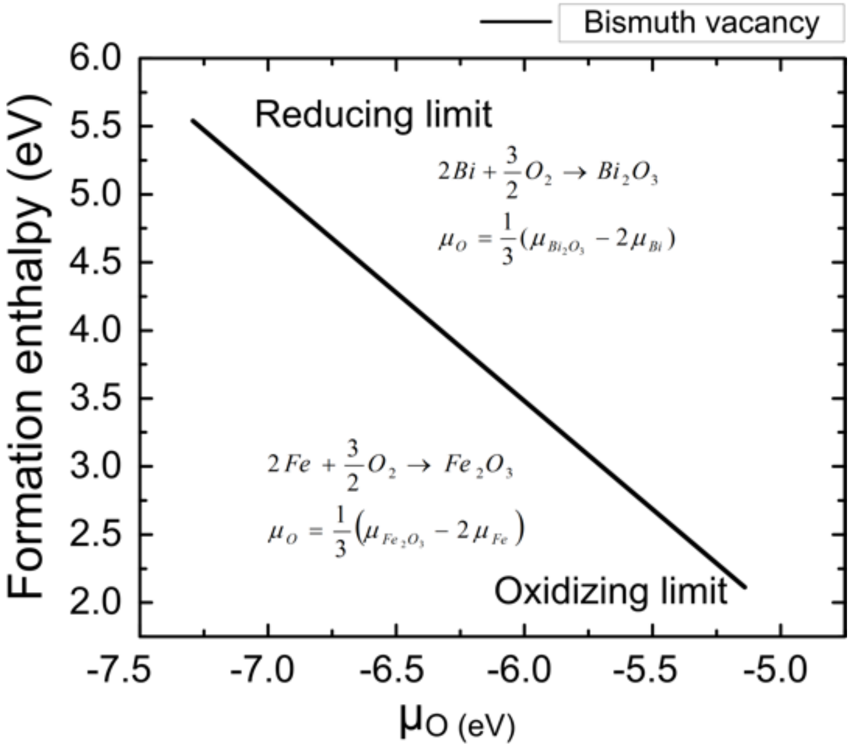

Figure S2. Formation enthalpy of Bi vacancy
